# Hyperlipidemia May Synergize with Hypomethylation in Establishing Trained Immunity and Promoting Inflammation in NASH and NAFLD

**DOI:** 10.1155/2021/3928323

**Published:** 2021-11-23

**Authors:** Charles I. V. Drummer, Fatma Saaoud, Yu Sun, Diana Atar, Keman Xu, Yifan Lu, Ying Shao, Candice Johnson, Lu Liu, Huimin Shen, Nirag C. Jhala, Xiaohua Jiang, Hong Wang, Xiaofeng Yang

**Affiliations:** ^1^Centers for Cardiovascular Research and Inflammation, Translational and Clinical Lung Research, Lewis Katz School of Medicine at Temple University, Philadelphia, PA 19140, USA; ^2^Metabolic Disease Research and Thrombosis Research, Department of Cardiovascular Sciences, Lewis Katz School of Medicine at Temple University, Philadelphia, PA 19140, USA; ^3^Department of Pathology, Lewis Katz School of Medicine at Temple University, Philadelphia, PA 19140, USA

## Abstract

We performed a panoramic analysis on both human nonalcoholic steatohepatitis (NASH) microarray data and microarray/RNA-seq data from various mouse models of nonalcoholic fatty liver disease NASH/NAFLD with total 4249 genes examined and made the following findings: (*i*) human NASH and NAFLD mouse models upregulate both cytokines and chemokines; (*ii*) pathway analysis indicated that human NASH can be classified into metabolic and immune NASH; methionine- and choline-deficient (MCD)+high-fat diet (HFD), glycine N-methyltransferase deficient (GNMT-KO), methionine adenosyltransferase 1A deficient (MAT1A-KO), and HFCD (high-fat-cholesterol diet) can be classified into inflammatory, SAM accumulation, cholesterol/mevalonate, and LXR/RXR-fatty acid *β*-oxidation NAFLD, respectively; (*iii*) canonical and noncanonical inflammasomes play differential roles in the pathogenesis of NASH/NAFLD; (*iv*) trained immunity (TI) enzymes are significantly upregulated in NASH/NAFLD; HFCD upregulates TI enzymes more than cytokines, chemokines, and inflammasome regulators; (*v*) the MCD+HFD is a model with the upregulation of proinflammatory cytokines and canonical and noncanonical inflammasomes; however, the HFCD is a model with upregulation of TI enzymes and lipid peroxidation enzymes; and (*vi*) caspase-11 and caspase-1 act as upstream master regulators, which partially upregulate the expressions of cytokines, chemokines, canonical and noncanonical inflammasome pathway regulators, TI enzymes, and lipid peroxidation enzymes. Our findings provide novel insights on the synergies between hyperlipidemia and hypomethylation in establishing TI and promoting inflammation in NASH and NAFLD progression and novel targets for future therapeutic interventions for NASH and NAFLD, metabolic diseases, transplantation, and cancers.

## 1. Introduction

Several metabolic diseases significantly drive the development of cardiovascular disease, nonalcoholic fatty liver disease (NAFLD) [1], and nonalcoholic steatohepatitis (NASH) [2]. NASH is characterized by macrovascular steatosis, hepatocellular ballooning, lobular inflammation, and pericellular fibrosis. NAFLD constitutes a major health concern, and NAFLD prevalence is estimated between 25% and 30% in the general population [3]. However, the exact underlying inflammatory mechanisms that trigger the transition from fatty liver to NASH remain unclear.

Innate immune system inflammation is considered a landmark of NASH pathogenesis. Mouse models of NASH have demonstrated that increased danger/pathogen-associated molecular pattern (DAMP/PAMP) receptor and Toll-like receptor (TLR) signaling [4] are associated with disease progression. Moreover, an intracellular DAMP/PAMP receptor and the inflammasome serve as a metabolic stress sensor and bridge metabolic distress to the initiation of pyroptosis, a proinflammatory programmed cell death [4–7]. In addition, inflammasomes serve as sensors of metabolic stresses for vascular, liver, and other inflammations [5, 6, 8–15]. Inflammasome pathways can be classified into the canonical caspase-1 or the noncanonical caspase-11-dependent inflammasome pathways [16]. The canonical inflammasome pathway has been shown to play a significant role in NASH and NAFLD pathogenesis [17, 18]. In addition to the molecular mechanisms of innate immunity, cellular mechanisms also play a significant role in NASH development.

The secretome can be defined as the portion of total protein secreted by cells to the extracellular space to maintain tissue homeostasis [19–22]. Treg may use innate immunity-related secretomes such as caspase-1-gasdermin D- (GSDMD-) dependent secretome [23] and caspase-4 (humans)/caspase-11 (mice)-GSDMD-dependent secretome [24] to carry out their functions. However, it remains unknown whether all the cytokines and chemokines in canonically and noncanonically defined secretomes play roles in the progression of NAFLD [25].

Innate immune cells can develop an exacerbated immunologic responses and long-term inflammatory phenotypes following brief exposure to endogenous or exogenous insults (the first challenge), which leads to an enhanced inflammatory response after a second challenge, which is known as trained immunity (TI) [4, 26–28]. TI is not only important for host defense and vaccine response but also for chronic inflammatory processes such as cardiovascular inflammation, EC activation [26, 27, 29], and liver injuries [30]. However, the role TI plays in the development of NASH/NAFLD has yet to be determined.

Oxidation of cholesterol and phospholipids can lead to lipid peroxidation and cell death [31–35]. Furthermore, lipid peroxidation drives canonical and noncanonical inflammasome-GSDMD-mediated pyroptosis via a glutathione peroxidase 4- (GPX4-) suppressed mechanism [33]. GPX4 is highly expressed in the liver [36], and hepatic ferroptosis [37] initiates inflammation in NASH via GPX4-suppressed manner [38]. However, the role of differential lipid peroxidation enzyme expression in NASH/NAFLD progression remains unclear.

DNA methylation plays a significant role in the progression of NAFLD. Previous studies have shown that there are differences in DNA methylation levels of multiple genes between liver samples from NAFLD patients with those from healthy individuals [39, 40], and further studies reveled that hypomethylation of different genes was more than the hypermethylation in advanced NAFLD versus mild NAFLD indicating that genes related to steatohepatitis, fibrosis, and tumorigenesis may be demethylated as NAFLD processes and the methylation levels of these genes may reflect the severity of NAFLD [41].

To fill in important knowledge gaps, we performed a panoramic database mining analysis on both human NASH microarray data and microarray data from various NAFLD mouse models and examined a total of 4249 genes by using the strategy we pioneered [9, 42, 43]. We made significant findings, which provide novel insights on the roles of proinflammatory cytokines and chemokines, canonical and noncanonical inflammasome pathways, TI enzymes, and lipid peroxidation in promoting NASH/NAFLD progression.

## 2. Materials and Methods

### 2.1. Expression profile of innatomic and secretomic genes, canonical and noncanonical inflammasome pathway regulators, trained immune genes, and lipid peroxidation genes in patients' nonalcoholic steatohepatitis (NASH) microarray data and microarray/RNA-seq data from various mouse models of nonalcoholic fatty liver disease

**Figure 1:**
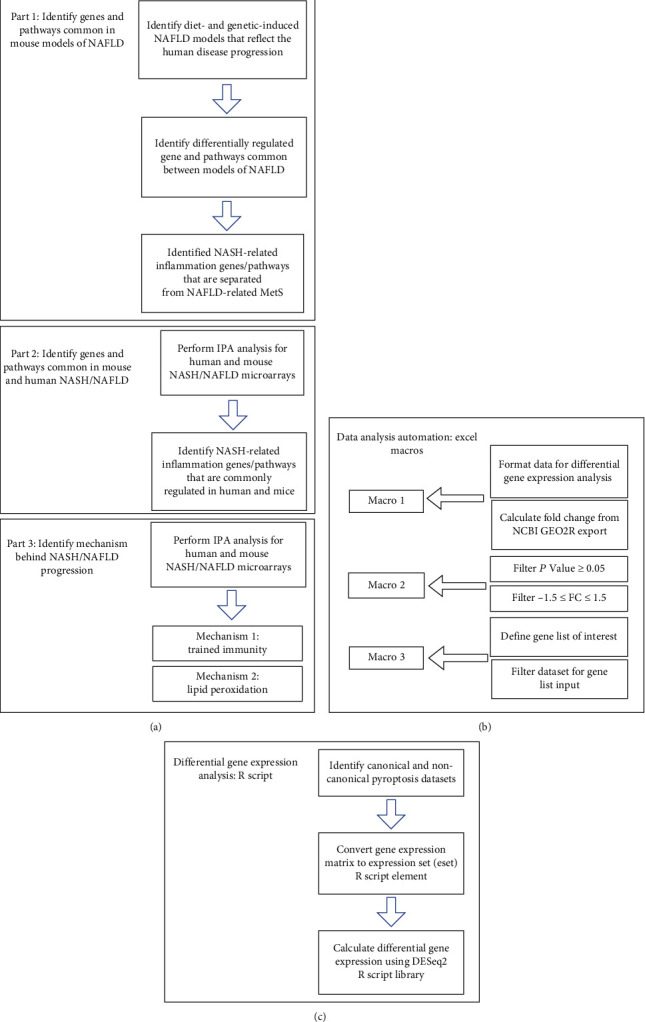
The flowchart of our data mining analyses. (a) Workflow included three parts: (1) identify genes and signaling pathways shared by microarrays from several mouse models of nonalcoholic fatty liver disease (NAFLD), (2) identify genes and signaling pathways common in mouse models of NAFLD and patients with nonalcoholic steatohepatitis (NASH), and (3) identify significant inflammatory mechanisms underlying the pathogenesis and progression of NASH/NAFLD. (b) Process automations using Microsoft Excel Macros, which have significantly facilitated the database mining processes comparing to that of our previous database mining papers. (c) GEO datasets without the GEO2R function were analyzed using DESeq2 library in R Studio. R script code used in C: Meeta Mistry, C. Titus Brown, jessicalumian, & tug65470 (2021, July 14), tug65470/msu_ngs2015: DESeq2 analysis of microarray and RNA-seq datasets (version v1.0.0), Zenodo, http://doi.org/10.5281/zenodo.5102949.

The 10 microarray datasets were collected from the National Institutes of Health- (NIH-) National Center for Biotechnology Information- (NCBI-) Gene Expression Omnibus (GEO) database and analyzed with an online software GEO2R (Figures 1(a) and 1(b)). One dataset was from the high-fat diet (HFD) and methionine- and choline-deficient (MCD) diet model (GSE35961). One dataset was from the high-fat-cholesterol diet- (HFCD-) induced diet model. One dataset was from the glycine N-methyltransferase- (GNMT-) KO genetic model, (4) the liver-specific methionine adenosyltransferase 1A- (MAT1A-) KO (GSE63027) genetic model. There were two human NASH microarrays (GSE63067 and GSE17470) [49]. For the mechanism studies, we collected one dataset from caspase-11-KO mice (GSE115094) (Figures 1(a) and 1(b)). One dataset was from caspase-1-KO mice (GSE32515). One human dataset was for trained immunity pathway (GSE24187). For datasets not formatted for GEO2R, analysis was performed using DESeq2 in R Studio as described by Mistry et al. (2015) (Figure 1(c)). In brief, the expression data obtained from NCBI GEO database was converted to an expression set R script element. Differential gene expression was analyzed using the DESeq2 library. The numbers and detailed information of the 7 GEO datasets are listed in Supplementary Tables 2A and 2B.

### 2.2. Ingenuity Pathway Analysis (IPA)

IPA was used to characterize the clinical relevance and molecular and cellular functions related to the identified genes. Differentially expressed genes were identified and uploaded into IPA for analysis. Core and canonical pathway analysis was used to identify molecular and cellular pathways [14, 15].

### 2.3. Statistical Analysis

We applied a statistical method similar to a meta-analysis [14, 27]. GEO dataset integrity used 7 housekeeping genes (Supplementary Tables 1 and 2) [44]. The target genes with expression changes more than 1.5-fold were defined as upregulated genes, while genes with their expression decreased more than 1.5-fold were defined as downregulated genes. Fold change calculations were performed in Excel as previously described by our group. Briefly, differential gene expression data were imported into Excel and then analyzed by four Excel Macros: (1) organize the DEG data from the DESeq2 and GEO2R, (2) filter data for significant *p* values, (3) filter data for fold change ± 1.5, and (4) retrieve expression data for various genes of interest (Figure 1(c)).

## 3. Results

### 3.1. Human NASH and NAFLD Mouse Models Upregulate Both Cytokines and Chemokines Classified in the Innate Immune Database and Canonical Secretome

At least 11 NAFLD mouse models have been established [45, 46] including diet-induced and genetic-induced models [47, 48], but no model accurately reflects the disease's progression in humans [3]. We include four mouse models that best represent the metabolic and inflammatory characteristics of human NAFLD [3, 46–48].

We hypothesized that diet- and genetically induced NAFLD mouse models can share common pathways critical to the development of the disease. To test this, we collected microarray datasets from NAFLD mouse models and human NASH (Supplementary Table 2a). The datasets we analyzed include (*1*) the high-fat diet (HFD) and methionine- and choline-deficient (MCD) diet model (GSE35961) [49], (*2*) the high-fat-cholesterol diet- (HFCD-) induced diet model [50], (*3*) the glycine N-methyltransferase- (GNMT-) KO genetic model, (*4*) the liver-specific methionine adenosyltransferase 1A- (MAT1A-) KO (GSE63027) [51] genetic model, and (*5*) two human NASH microarrays (GSE63067 [51] and GSE17470 [52]). The qualities of the datasets were assessed using a few housekeeping genes (Supplementary Tables 1a and 1b).

**Figure 2:**
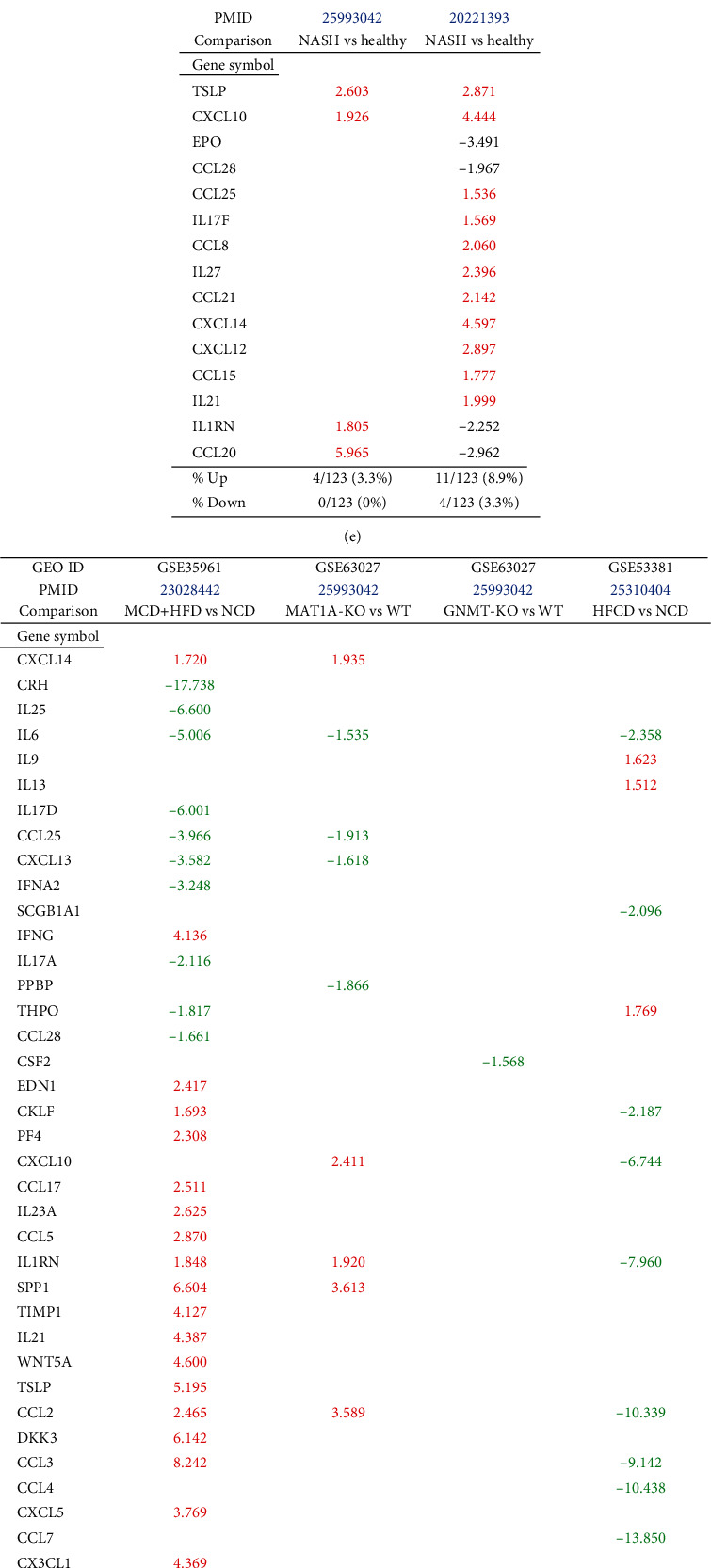
The cytokine and chemokine expressions are increased in both human nonalcoholic steatohepatitis (NASH) and mouse models of nonalcoholic fatty liver disease (NAFLD). Ingenuity Pathway Analysis results showed that 53 cytokines and chemokines were sorted out from 1376 innate immune genes (innatome, from the Innate Immune Database (https://www.innatedb.com/); also, see our paper (PMID: 33628851)) and 123 cytokines and chemokines were sorted out from 2641 canonical secretome (with signal peptide) genes (from the Human Protein Atlas database (https://www.proteinatlas.org/); also, see our paper (PMID: 32179051)). (a) A schematic figure showed the pathogenic effects of cytokines released from NASH on different organs. Two human NASH datasets (GSE63067 and GSE17470) and four mouse models of NAFLD datasets (GSE35961, GSE63027, GSE63027, and GSE53381) were analyzed. (b, c) The 53 innatome cytokines and chemokines were analyzed in human NASH and mouse models of NAFLD. (d) Venn diagram showed the significant overlapped regulated innatome cytokines and chemokines in human NASH and mouse models of NAFLD. The cytokine and chemokine expressions are increased in both human nonalcoholic steatohepatitis (NASH) and mouse models of nonalcoholic fatty liver disease (NAFLD). (e, f) The 123 canonical secretomic cytokines and chemokines were analyzed in human NASH and mouse models of NAFLD. (g) Venn diagram showed the overlapped significant regulated canonical secretomic cytokines and chemokines in human NASH and mouse models of NAFLD. The cytokine and chemokine expressions are increased in both human nonalcoholic steatohepatitis (NASH) and mouse models of nonalcoholic fatty liver disease (NAFLD). (h) The 16 cytokines and chemokines (innatome and secretome) were upregulated (upregulated in GSE63067 or GSE17470) in human NASH. PMID: 34084175. (i) The 16 cytokines and chemokines (innatome and secretome) were upregulated (GSE35961 or GSE63027) in mouse models of NAFLD. Secretome gene list detailed in our previous publication PMID: 34084175.

Cytokines and chemokines [53–55] play significant roles in promoting the onset and progression of NAFLD [55]; however, their expression changes in human NASH and NAFLD mouse models remained poorly characterized. Cytokines and chemokines in the liver are soluble mediators of local and systemic inflammation (Figure 2(a)) [8, 43, 56, 57]. To analyze liver inflammation, we used IPA and classified 53 cytokines and chemokines from 1376 innate immune genes [58, 59] from the Innate Immunity Database [58, 60]. Human NASH upregulated six (11.3%) and four (7.5%) out of 53 innate immune cytokines and chemokines, respectively (Figure 2(b)). Of note, upregulations of C-X-C motif chemokine ligand 10 (CXCL10) and CKLF-like MARVEL transmembrane domain-containing 2 (CMTM2) were shared in these two human NASH datasets. MCD+HFD and MAT1A-KO upregulated 13 (24.5%) and 6 (11.3%) out of 53 innate immune cytokines and chemokines, respectively (Figure 2(c)). MCD+HFD, MAT1A-KO, GNMT-KO, and HFCD downregulated 9 (17%), 3 (5.7%), 1 (1.9%), and 12 (22.6%) out of 53 innate immune cytokines and chemokines, respectively.

To determine whether NAFLD mouse models share innate immune cytokines and chemokines with human NASH, we performed Venn diagram analysis. Six innate immune cytokines and chemokines including CXCL10, tumor necrosis factor (ligand) superfamily, member 13b (TNFSF13B), lymphotoxin beta (LTB, TNFSF3), TNFSF10, interleukin 1 receptor antagonist (IL-1RN), and nicotinamide phosphoribosyltransferase (NAMPT) were shared between human NASH and NAFLD mouse models (Figure 2(d)). Two NAFLD mouse models upregulated 22 (78.6%) specific innate immune cytokines and chemokines; and human NASH upregulated two (25%) specific cytokines and chemokines C-C motif chemokine ligands 20 (CCL20) and CMTM2.

To ensure a comprehensive analysis of liver inflammation in human NASH and NAFLD mouse models, we collect 2641 canonical secretome genes [61]. The 123 cytokines and chemokines were classified with IPA out of 2641 human canonical secretome genes. Human NASH upregulated 4 (3.3%) and 11 (8.9%) out of 123 canonical secretomic cytokines and chemokines (Figure 2(e)). Of note, upregulations of CXCL10 and thymic stromal lymphopoietin (TSLP) were shared in the two human NASH datasets. MCD+HFD, MAT1A-KO, GNMT-KO, and HFCD models upregulated 21 (17.1%), 7 (5.7%), 0, and 3 (2.4%) (Figure 2(f)) and downregulated 10 (8.1%), 4 (3.3%), 1 (0.8%), and 10 (8.1%) out of 123 canonical secretomic cytokines and chemokines, respectively.

To determine whether NAFLD mouse models share canonical secretomic cytokines and chemokines with human NASH, we performed Venn diagram analysis. Seven canonical secretomic cytokines and chemokines including TSLP, CXCL10, CCL28, CCL25, CXCL14, IL21, and IL1RN were shared between NAFLD mouse models and human NASH (Figure 2(g)). By comparison, three NAFLD mouse models upregulated 32 (82.1%) specific canonical secretomic cytokines and chemokines; and human NASH upregulated 8 (53.3%) specific cytokines and chemokines including erythropoietin (EPO), IL17F, CCL8, IL27, CCL21, CXCL12, CCL15, and CCL20.

Caspase-1-deficient mice are protected from high fat-induced hepatic steatosis, inflammation, and early fibrogenesis [62], suggesting that pyroptosis-related caspase-1/inflammasomes [6] promote NASH progression. However, an important question remains whether noncanonical inflammasome pathway-related caspase-4/11 [63] plays any role in progression of NAFLD.

To determine whether NASH/NAFLD upregulated cytokines and chemokines classified into noncanonical secretomes, we collected four types of secretomes with total 11,385 proteins including canonical secretome (2641 proteins) [61], caspase-1-dependent noncanonical secretome (961 proteins) [23], caspase-4-dependent noncanonical secretome (1223 proteins) [24], and exosome secretome (6560 proteins) [25]. We combined human NASH and NAFLD mouse upregulated cytokines and chemokines and performed Venn diagram analysis. Among 15 human NASH-upregulated cytokines and chemokines, two cytokines CMTM2 and LTB were nonclassified, 12 out of 15 (80%) were classified in the canonical secretome, TNFSF10 was classified in canonical and exosome secretomes, and NAMPT was classified in canonical and caspase-4 secretomes (Figure 2(h)). Of note, since 123 cytokines and chemokines identified with the IPA are derived from the canonical secretome, it was expected that the high percentage (80%) NASH-upregulated cytokines and chemokines were from the canonical secretome. Among 26 NAFLD mouse-upregulated cytokines and chemokines, one was the nonclassified, 15 were classified in the canonical secretome, seven were classified in canonical and exosome secretomes, NAMPT was classified in canonical and caspase-4 secretomes, and two cytokines were classified in canonical, exosomes, and caspase-4 secretomes (Figure 2(i)).

Taken together, our results have demonstrated innate immune secretomic cytokines and chemokines suggesting that human NASH and NAFLD mouse models share innate immune mechanisms and there is a significant role of exosomes and caspase-4 secretomic in driving liver and systemic inflammations.

### 3.2. Human NASH Can Be Classified into Metabolic and Immune NASH; MCD+HFD, GNMT-KO, MAT1A-KO, and HFCD Can Be Classified into Inflammatory, SAM Accumulation, Cholesterol/Mevalonate, and LXR/RXR-Fatty Acid *β*-Oxidation NAFLD, Respectively

To determine the signaling pathways mediating the transcriptomic changes in human NASH and NAFLD mouse models, we performed IPA analysis for the top 20 pathways for significantly modulated genes in each dataset. Human NASH dataset GSE17470 had top upregulated pathways including phospholipases, liver X receptor (LXR)/retinoid X receptor (RXR) activation, fatty acid *β*-oxidation I, and leukocyte extravasation, among which leukocyte extravasation was shared by the second human NASH and MCD+HFD, LXR/RXR activation was shared by HFCD, and fatty acid *β*-oxidation was shared by GNMT-KO and HFCD (Table 1, Supplementary Figure 1).

The GNMT-KO (GSE63027) had top upregulated pathways including fatty acid *β*-oxidation I, stearate biosynthesis I, antioxidant action of vitamin C, oxidative phosphorylation, glutaryl-CoA degradation kinase, triacylglycerol biosynthesis, and tricarboxylic acid cycle (TCA) cycle II, among which fatty acid *β*-oxidation I and stearate biosynthesis I were shared with the first human NASH (GSE17470). The MAT1A-KO (GSE63027) had top upregulated pathways including nicotine degradation II, superpathway of cholesterol biosynthesis, cholesterol biosynthesis I, cholesterol biosynthesis III, mevalonate pathway I, and NAD salvage pathway II, among which nicotine degradation II was shared with the first human NASH.

The HFCD model (GSE53381) has only four upregulated pathways including LXR/RXR activation and fatty acid *β*-oxidation I, among which LXR/RXR activation was shared with the first human NASH, fatty acid *β*-oxidation II was shared with the first human NASH and GNMT-KO, and nicotine degradation II was shared with MAT1A-KO.

The second human NASH (GSE63067) had top upregulated pathways including NF-*κ*B signaling and neuroinflammation (Table 1). The MCD+HFD model (GSE35961) had top upregulated pathways including leukocyte extravasation, antibody Fc fragment gamma (Fc*γ*) receptor-mediated phagocytosis, IL-8 signaling, Rac signaling, and glucose 6-phosphate (G6P) signaling, among which IL-8 signaling was shared with the second human NASH.

Taken together, our results have demonstrated that the two human NASH datasets have diversified signaling pathways, which allow us to classify the first one into LRX/RXR activation-related metabolic NASH and the second NASH into NF-*κ*B signaling [64]/T helper cell 17 (Th17) [56, 65] inflammatory NASH. IPA of upregulated genes in each mouse models of NASH and human NASH identified top pathways for significantly modulated genes. The major differences between metabolic NASH and immune NASH include the following: first, numerous inflammation and immune pathways are identified on the top pathway list, and second, metabolic reprogramming in any given mouse models affects four metabolic pathways including (1) increased glycolysis, (ii) glutaminolysis, (iii) increased accumulation of tricarboxylic acid cycle metabolites and acetyl-coenzyme A production, and (iv) increased mevalonate synthesis. Furthermore, this classification provides a criterion for mouse model stratification and selection, providing novel insight of which mouse models best represent various human cases of NASH/NAFLD.

### 3.3. Canonical and Noncanonical Inflammasome Pathways Play Differential Roles in the Pathogenesis of NASH/NAFLD

To examine the differential roles of caspase-1- and caspase-11-dependent pyroptosis, we collected 90 canonical and 14 noncanonical inflammasome pathway regulator genes and determined the expression changes of these inflammasome regulators in four NAFLD mouse models.

**Figure 3:**
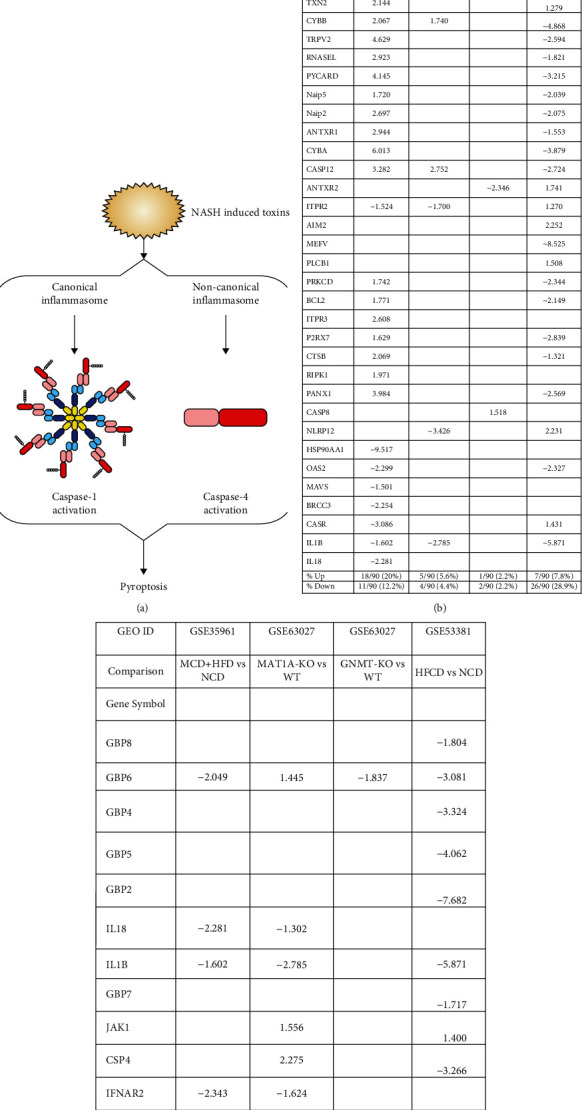
Mouse pyroptosis pathway regulators. (a) Schematic representation of canonical vs. noncanonical pyroptosis. (b) Mouse canonical inflammasome pathway regulators (90 genes, KEGG pathway hsa04621) were differentially expressed in NASH/NAFLD models. The MCD+HFD model upregulated 18 out of 90 (20%) canonical inflammasome pathway regulators including caspase-1 and downregulated 11 out of 90 (12.2%) canonical inflammasome pathway regulators. The MAT1A-KO model upregulated 5 out of 90 (5.6%) and downregulated 4 out of 90 (4.4%) canonical inflammasome pathway regulators. The GNMT-KO model upregulated 1 out of 90 (1.1%) and downregulated 2 out of 90 (2.2%) canonical inflammasome pathway regulators. The HFCD model upregulated 7 out of 90 (7.8%) and downregulated 26 out of 90 (28.9%) canonical inflammasome pathway regulators. (c) Mouse noncanonical inflammasome pathway regulators (14 genes, KEGG pathway hsa04621) were differentially expressed in NASH/NAFLD. The MCD+HFD model upregulated zero out of 14 (0%) and downregulated 6 out of 14 (42.8%) noncanonical inflammasome pathway regulators. The MAT1A-KO model upregulated 3 out of 14 (21.4%) and downregulated 3 out of 14 (21.4%) noncanonical inflammasome pathway regulators. The GNMT-KO model upregulated 0 out of 14 (0%) and downregulated 1 out of 14 (7.1%) noncanonical inflammasome pathway regulators. The HFCD model upregulated 1 out of 14 (7.1%) and downregulated 9 out of 14 (64.3%) noncanonical inflammasome pathway regulators.

MCD+HFD upregulated 18 (20%) including caspase-1 and downregulated 11 (12.2%) out of 90 canonical inflammasome pathway regulators. MAT1A-KO upregulated 5 (5.6%) and downregulated 4 (4.4%) out of 90 canonical inflammasome pathway regulators. GNMT-KO upregulated 1 (1.1%) and downregulated 2 (2.2%) out of 90 canonical inflammasome pathway regulators. HFCD upregulated 7 (7.8%) and downregulated 26 (28.9%) out of 90 canonical inflammasome pathway regulators (Figure 3(b)). In addition, MCD+HFD downregulated 6 out of 14 (42.8%) noncanonical inflammasome pathway regulators. MAT1A-KO upregulated 3 (21.4%) and downregulated 3 (21.4%) out of 14 noncanonical inflammasome pathway regulators. GNMT-KO downregulated 1 out of 14 (7.1%) noncanonical inflammasome pathway regulators. HFCD upregulated 1 (7.1%) and downregulated 9 (64.3%) out of 14 noncanonical inflammasome pathway regulators (Figure 3(c)).

Taken together, these results have demonstrated that the MCD+HFD upregulates more canonical inflammasome regulators than the other three NAFLD models. Conversely, the MAT1A-KO upregulates more noncanonical inflammasome regulators including caspase-4 than other models, suggesting that canonical and noncanonical inflammasome pathways are regulated differentially in various NAFLD models. The HFCD downregulates more than upregulates canonical and noncanonical inflammasome regulators, suggesting that HFCD model uses more focused set of regulators in modulating the expression of these regulators.

### 3.4. Trained Immunity (TI) Enzymes Are Significantly Upregulated in Human NASH and NAFLD Mouse Models; the HFD Upregulates TI Enzymes More Than Cytokines, Chemokines, and Canonical and Noncanonical Inflammasome Regulators; and Statins Promote rather than Suppress TI Enzyme Expressions

**Figure 4:**
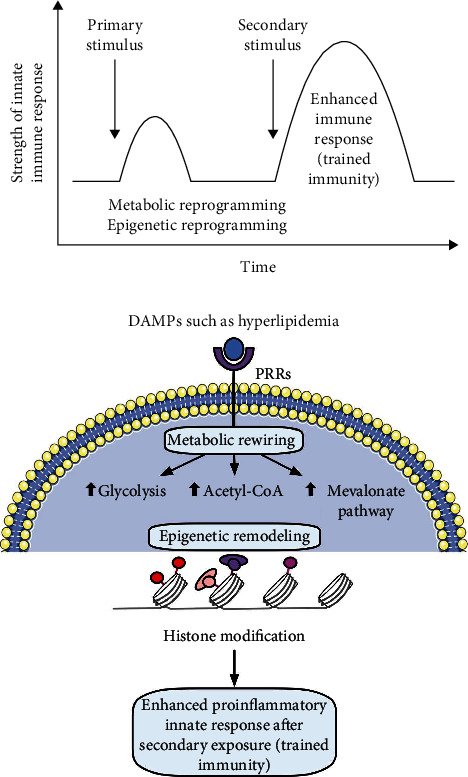
Schematic representation of fundamental mechanisms in trained immunity. Danger-associated molecular patterns (DAMPs) can bind to their corresponding pattern recognition receptors (PRRs) and alter metabolic pathways including increased glycolysis, increased acetyl-CoA generation, and mevalonate synthesis in cholesterol pathway, leading to histone modifications that enable chromatin regions to be more open for transcription. Increased gene expression of proinflammatory cytokines/chemokines and enhanced proinflammatory innate immune response against pathogens during secondary exposure. Figure created with Smart Servier Medical Art and Omnigraffle.

We hypothesized that the expressions of TI enzymes are modulated in NASH and NAFLD to establish metabolic reprogramming and achieve innate immune memory [66]. Three metabolic pathways play significant roles in establishing TI (Figure 4). To examine this, we collected 71 glycolysis enzyme genes, 24 acetyl-CoA generation enzyme genes, and 10 mevalonate genes [27, 29, 30, 67, 68].

The first human NASH upregulated 25 (35.2%) and downregulated 5 (7%) out of 71 glycolysis enzymes genes (Table 2(a)). The second human NASH upregulated 5 out of 71 (7%) genes. The MCD+HFD upregulated 20 (28.2%) and downregulated 24 (33.8%) out of 71 genes. The MAT1A-KO upregulated 6 (8.5%) and downregulated 13 (18.3%) out of 71 genes. The GNMT-KO model upregulated 7 (9.9%) and downregulated 4 (5.6%) out of 71 genes. The HFCD upregulated 17 (23.9%) and downregulated 8 (11.3%) out of 71 genes.

Furthermore, the first human NASH upregulated 4 out of 24 (16.6%) acetyl-CoA generation enzyme genes. The second human NASH upregulated 1 out of 24 (4.2%) genes. The MCD+HFD upregulated 3 (12.5%) and downregulated 7 (29.2%) out of 24 genes. The MAT1A-KO upregulated 1 (4.2%) and downregulated 4 (16.7%) out of 24 genes. The HFCD upregulated 7 (29.2%) and downregulated 2 (8.3%) out of 24 genes (Table 2(b)).

In addition, the first human NASH upregulated 2 (20%) and downregulated 3 (30%) out of 10 mevalonate pathway enzyme genes. The second human NASH upregulated 1 out of 10 (10%) gene. The MCD+HFD upregulated 7 (70%) and downregulated 1 (10%) out of 10 genes. The MAT1A-KO upregulated 4 (40%) and downregulated 1 (10%) out of 10 genes. The GNMT-KO upregulated 3 out of 10 (30%) genes. The HFCD upregulated 7 out of 10 (70%) genes (Table 2(c)).

Statins (*β*-hydroxy *β*-methylglutaryl-CoA (HMG-CoA) reductase inhibitors) block cholesterol synthesis and mevalonate generation, preventing TI induction [69]; and statin treatments do not revert the TI phenotype in patients with familiar hypercholesterolemia [70]. We hypothesized that statins may modulate TI enzyme expression in NAFLD models. For this, we collected two microarray datasets of statin-treated human primary hepatocytes [71].

**Figure 5:**
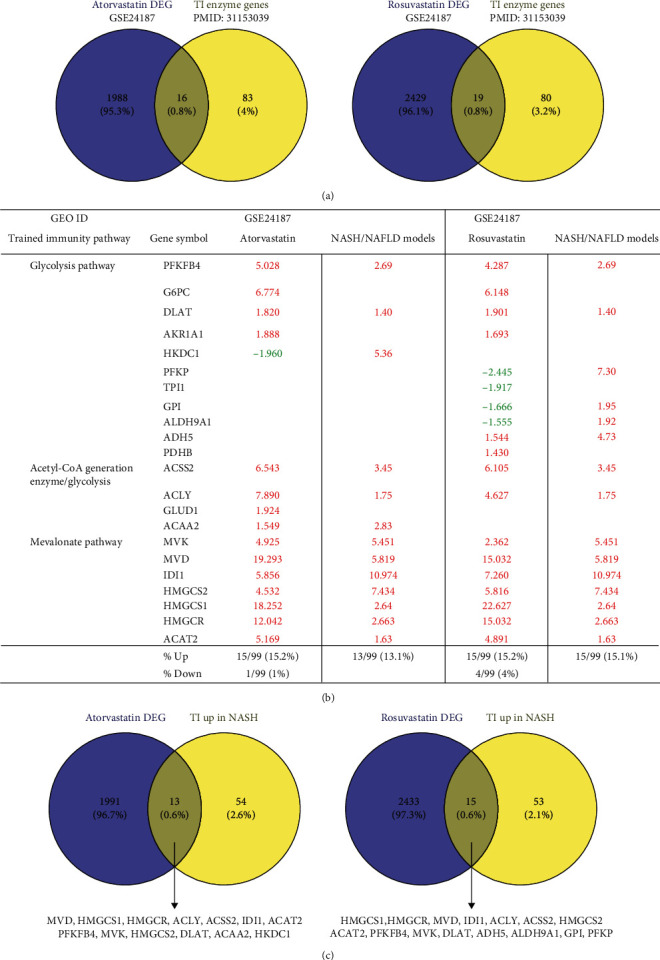
Trained immunity enzyme genes were differentially expressed in statin treatment (HMG-CoA reductase inhibitor) in the mevalonate synthesis pathway and modulated in human and mouse NASH/NAFLD. (a) Venn diagram showed the overlapped trained immunity enzyme genes (PMID: 31153039) with differentially expressed genes (DEG) in statin (atorvastatin and rosuvastatin) treatment (GSE24187). (b) List of trained immunity enzyme genes differentially expressed in atorvastatin and rosuvastatin and upregulated in human and mouse NASH/NAFLD models. (c) Venn diagram showed that the upregulated trained immunity enzyme genes in human and mouse NASH/NAFLD overlapped with the differentially expressed genes in atorvastatin and rosuvastatin treatment.

Among the 2004 differentially expressed genes, 16 genes were overlapped with 99 TI enzyme genes (16.2%) [27], of which 15 (15.2%) genes were upregulated and one (1%) gene was downregulated by atorvastatin (Figures 5(a)–5(c)). In addition, among 2448 differentially expressed genes in rosuvastatin-treated human primary hepatocytes, 19 genes were overlapped with 99 TI enzyme genes (19.2%), of which 15 (15.2%) genes were upregulated and 4 (4%) genes were downregulated by rosuvastatin. Then, we used Venn diagram to determine the causative effects of statins in TI enzyme genes upregulated in NAFLD models. The results showed that the atorvastatin treatment overlapped with 13 TI enzyme genes upregulated in human and mouse NASH/NAFLD models. Similarly, rosuvastatin treatment overlapped with 15 TI enzyme genes upregulated in human and mouse NAFLD models. These results have demonstrated that TI inhibitors, statins, promote the expression of TI enzyme genes not only in mevalonate pathways but also in glycolysis pathways and acetyl-CoA generation pathway.

### 3.5. Expression Analysis of Lipid Peroxidation Enzymes Indicates That There Are Two Representative NASH/NAFLD Models: The MCD+HFD Model Is Proinflammatory Cytokine- and Canonical and Noncanonical Inflammasome-Upregulated Model; in Contrast, the HFCD Model Is Lipid Peroxidation Enzyme- and TI Enzyme-Upregulated Model

**Figure 6:**
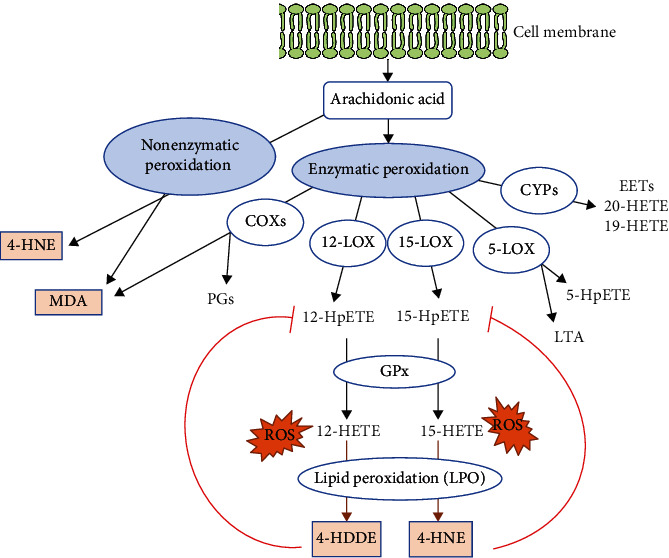
Schematic representation of arachidonic acid metabolism and lipid peroxidation. Cyclooxygenase (COX), lipoxygenase (LOX), and cytochrome p450s (CYPs) mediated enzymatic peroxidation of arachidonic acid (AA) to the respective metabolites. The 12-lipoxygenase (12-LO) and 15-lipoxygenase (15-LO) mediated conversion of AA to 12- and 15-hydroperoxyeicosatetraenoic acid (HpETE) and the downstream glutathione peroxidase- (GPx-) mediated conversion to the respective hydroxyeicosatetraenoic acids (12- and 15-HETE). Reactive oxygen species- (ROS-) induced lipid peroxidation (LPO) of 12- and 15-HpETE results in the generation of 4-hydroxynonenal (4-HNE) and 4-hydroxydodecadeinal (4-HDDE). These reactive aldehydes interact with and inactivate GPx, leading to an increased rate of 12-HpETE and 15-HpETE peroxidation (PMID: 29610056). The nonenzymatic peroxidation mediates conversion of AA to 4-HNE and MDA metabolites (PMID: 24999379).

Arachidonic acid (AA) undergoes oxidative metabolism under enzymatic or nonenzymatic mechanisms. Cyclooxygenase (COX), lipoxygenase (LOX), and cytochrome p450s (CYPs) mediated enzymatic peroxidation of AA to the respective metabolites. The 12-lipoxygenase (12-LO) and 15-lipoxygenase (15-LO) mediated conversion of AA to 12- and 15-hydroperoxyeicosatetraenoic acid (HpETE) and the downstream glutathione peroxidase- (GPx-) mediated conversion to the respective hydroxyeicosatetraenoic acids (12- and 15-HETE). ROS-induced lipid peroxidation (LPO) of 12- and 15-HpETE results in the generation of 4-hydroxynonenal (4-HNE) and 4-hydroxydodecadeinal (4-HDDE). These reactive aldehydes interact with and inactivate GPx, leading to an increased rate of 12-HpETE and 15-HpETE peroxidation [72]. The nonenzymatic peroxidation mediates conversion of AA to 4-HNE and MDA metabolites [73] (Figure 6).

We hypothesized that downregulation of lipid peroxidation antioxidant enzymes CYPs and GPXs is inversely associated with upregulation of cytokines and chemokines in NAFLD and that upregulation of proinflammatory lipid peroxidation enzymes COXs and LOXs [74] is associated with upregulations of TI enzymes in NAFLD. To examine these hypotheses, we collected 26 COX lipid peroxidation enzymes, 44 cytochrome P450 CYP lipid peroxidation antioxidant (anti-inflammatory) enzymes [75, 76], 26 AA metabolism enzymes, and 8 hepatic glutathione peroxidase antioxidant (anti-inflammatory) enzymes [77].

The MCD+HFD upregulated 2 (7.7%) and downregulated 9 (34.6%) COXs (Table 3(a)). The MAT1A-KO upregulated 2 (7.7%) and downregulated 1 (3.8%) out of 26 COXs. The GNMT-KO upregulated 3 (11.5%) COXs. The HFCD upregulated 9 (34.6%) and downregulated 1 (3.8%) COXs.

The MCD+HFD upregulated 6 (13.6%) and downregulated 13 (29.5%) CYP lipid peroxidation antioxidant enzymes (CYP-LPAEs) (Table 3(c)). The MAT1A-KO upregulated 4 (9.1%) and downregulated 8 (18.2%) CYP-LPAEs. The GNMT-KO upregulated 5 (11.4%) and downregulated 8 (18.2%) CYP-LPAEs. The HFCD upregulated 22 (50%) and downregulated 1 (2.3%) CYP-LPAEs (Figure 6(c)).

The MCD+HFD upregulated 4 (15.4%) and downregulated 11 (42.3%) AA metabolism enzyme genes (Table 3(d)). The MAT1A-KO upregulated 4 (15.4%) and downregulated 6 (23.1%) genes. The GNMT-KO upregulated 4 (15.4%) and downregulated 7 (26.9%) genes. The HFCD upregulated 24 (92.3%) genes (Figure 6(d)).

The MCD+HFD upregulated 4 out of 8 (50%) GPXs (Table 3(b)). The MAT1A-KO upregulated 2 (25%) GPXs. The GNMT-KO upregulated 1 out of 8 (12.5%) GPXs. The HFCD downregulated 1 out of 8 (12.5%) GPXs (Figure 6(e)).

Taken together, our results have demonstrated that, *first*, proinflammatory MCD+HFD upregulates 7.7% COXs, 13.6% CYP-LPAEs, 15.4% AA metabolism enzymes, and 50% GPXs and downregulates 34.6% COXs, 29.5% CYP-LPAEs, and 42.3% AA metabolism enzymes, which are in big contrast with that of less inflammatory HFCD with upregulations of 34.6% COXs, 50% CYP-LPAEs, and 92.3% AA metabolism enzymes and downregulation of 3.8% COXs, 2.3% CYP-LPAEs, and 12.5% GPXs; *second*, significant upregulation of lipid peroxidation enzymes in HFCD is positively correlated with significant upregulation of TI enzymes and is, however, inversely associated with low-level upregulation of canonical inflammasome regulators (6.7%), noncanonical inflammasome regulators (7.1%), and 2.4% secretomic cytokines; *third*, no upregulation of GPXs in HFCD (Table 3(b)) is well correlated with other's findings that GPX4 decreases lipid peroxidation and inflammation, and caspase-11-dependent pyroptosis mediates atherosclerosis [78], endotoxic shock [79], and septic death in GPX4-KO mice [33]; and *fourth*, the MCD+HFD and HFCD are the two representative models: the MCD+HFD is a model of NASH/NAFLD with upregulation of proinflammatory cytokines and canonical and noncanonical inflammasome regulators. In contrast, the HFCD is a model with upregulation of lipid peroxidation enzymes and TI enzymes.

### 3.6. As Upstream Master Regulators, Caspase-11 and Caspase-1 Partially Upregulate the Expressions of Cytokines, Chemokines, Canonical and Noncanonical Inflammasome Pathway Regulators, TI Enzymes, and Lipid Peroxidation Enzymes

It has been reported that caspase-11 mediates hepatocyte pyroptosis and promotes the progression of MCD diet-induced NASH/NAFLD [80]; and inflammasome-gasdermin D-pyroptosis [81] may regulate the progression of NASH/NAFLD [82]. In addition to promoting inflammation by enzymatic cleavage of pre-IL-1*β*, pre-IL-18, and N-terminal GSDMD, caspase-1 also cleaves as many as 114 substrates, suggesting that caspase-1 may regulate inflammation indirectly via all the protein substrate-mediated signaling [15], 38 interaction protein-mediated signaling [15], and GSDMD-secretome-mediated signaling [23]. However, an important question remains whether caspase-11 modulates the expression of cytokines, chemokines, and TI enzymes. We hypothesized that caspase-11 promotes inflammatory cytokines and chemokines upregulated in NASH/NAFLD and modulates the expression of canonical and noncanonical inflammasome pathway genes and TI enzymes. To examine this hypothesis, we collected all the cytokines and chemokines, canonical and noncanonical inflammasome pathway genes, and TI enzymes upregulated in NASH/NAFLD and crossed with caspase-11-KO and caspase-1-KO datasets (Supplementary Table 2b).

Among 39 innatomic and secretomic cytokines and chemokines upregulated in NASH/NAFLD, caspase-11 deficiency upregulated 18 (46.2%) and downregulated 8 (20.5%) NASH/NAFLD-upregulated cytokines and chemokines. In comparison, caspase-1 deficiency upregulated one cytokine CXCL14 (2.6%) and downregulated one cytokine LTB (2.6%) NASH/NAFLD-upregulated cytokines and chemokines (Table 4(a)). These results suggest that capsase-11 deficiency promotes the upregulation of most cytokines and chemokines induced by NASH/NAFLD. In addition, caspase-11 deficiency upregulated 5 (17.9%) and downregulated 5 (17.9%) canonical inflammasome regulators induced by NASH/NAFLD. However, caspase-1 deficiency did not modulate the expression of canonical inflammasome regulators induced by NASH/NAFLD (Table 4(b)). Furthermore, caspase-11 deficiency upregulated 6 (8.8%) and downregulated 31 (45.6%) TI enzymes upregulated in NASH/NAFLD. In comparison, caspase-1 deficiency upregulated 4 (5.9%) and downregulated 6 (8.8%) TI enzymes upregulated in NASH/NAFLD (Table 4(c)). Finally, caspase-11 deficiency upregulated 13 (28.3%) and downregulated 6 (13%) lipid peroxidation enzymes upregulated in NASH/NAFLD. In comparison, caspase-1 deficiency upregulated 3 (6.5%) and downregulated 6 (13%) lipid peroxidation enzymes upregulated in NASH/NAFLD (Table 4(d)).

Taken together, our results have demonstrated that, *first*, caspase-11 and caspase-1 partially modulate the expressions of cytokines, chemokines, canonical and noncanonical inflammasome pathway regulators, TI enzymes, and lipid peroxidation enzymes; *second*, in the context of NASH/NAFLD, caspase-11 has stronger capacities than caspase-1 in modulating inflammatory gene expressions; and *third*, experimental conditions in caspase-11 deficiency [79] and caspase-1 deficiency [83] were not exactly the same as NASH/NAFLD. Since we used inflammatory gene lists upregulated in the four NASH/NAFLD models for the analyses, our results are at least partially relevant to NASH/NAFLD pathology. Future work on transcriptomic analysis of caspase-11 deficiency and caspase-1 deficiency in NASH/NAFLD models, respectively, is needed to verify these findings presented here.

## 4. Discussion

In this study, we performed a panoramic database mining analysis on microarray data of both human NASH and NAFLD mouse models. We made the following significant findings: (*i*) human NASH and NAFLD mouse models upregulate both cytokines and chemokines and canonical secretome; (*ii*) pathway analysis indicated that human NASH can be classified into metabolic and immune NASH; MCD+HFD, GNMT-KO, MAT1A-KO, and HFCD can be classified into inflammatory NAFLD, SAM accumulation NAFLD, cholesterol/mevalonate NAFLD, and LXR/RXR-fatty acid *β*-oxidation NAFLD, respectively; (*iii*) canonical and noncanonical inflammasome pathways play differential roles in the pathogenesis of NASH/NAFLD; (*iv*) TI enzymes are significantly upregulated in human NASH and NAFLD mouse models; HFD upregulates TI enzymes more than cytokines, chemokines, and canonical and noncanonical inflammasome regulators; statins promote rather than suppress TI enzyme expression; (*v*) lipid peroxidation enzyme expression indicated that there are two representative NAFLD models: the MCD+HFD is a proinflammatory cytokine- and canonical and noncanonical inflammasome-upregulated model; in contrast, the HFCD is a lipid peroxidation enzyme- and TI enzyme-upregulated model; and (*vi*) caspase-11 and caspase-1 partially upregulate the expressions of cytokines, chemokines, canonical and noncanonical inflammasome pathway regulators, TI enzymes, and lipid peroxidation enzymes.

**Figure 7:**
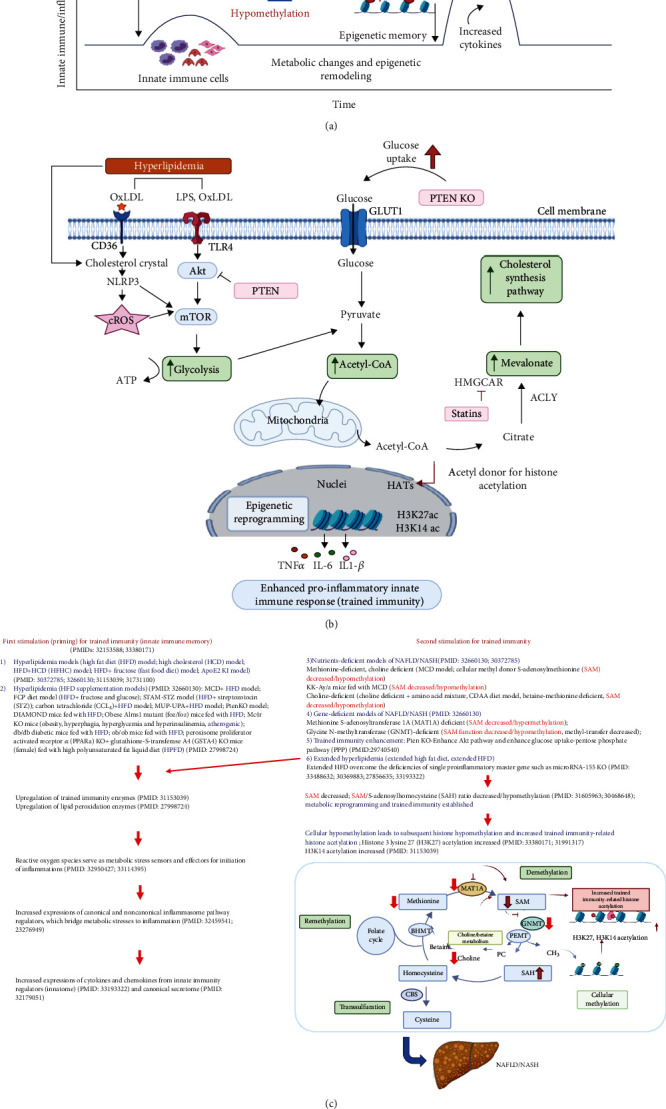
Our new integrated working model: multiple-hit trained immunity model with hyperlipidemia and hypomethylation (S-adenosylmethionine (SAM) decrease) for inflammation enhancement in various diet-induced and gene-deficient/genetic mouse models of nonalcoholic steatohepatitis (NASH)/nonalcoholic fatty liver disease (NAFLD). (a) Schematic presentation of trained immunity in NASH and NAFLD. Hyperlipidemia (high-fat diet) acts as the first stimuli to prime the innate immune cells and induce trained immunity. Hypomethylation that is caused by methionine-related nutrient deficiency and SAM synthase deficiency is regarded as second stimulation. This restimulation establishes trained immunity and metabolic reprogramming, leading to a large number of inflammatory cytokine secretion. (b) Epigenetic reprogramming pathway of trained immunity: hyperlipidemia and hyperglycemia inducers bind to their receptors in the cell membrane, increasing trained immunity-related glycolysis, acetyl-CoA production, and mevalonate pathways. Upregulation of three trained immunity pathways leads to increased trained immunity-related histone acetylation (H3K27ac and H3K14ac) and enhance proinflammatory innate immune response. (c) Overview figure shows the trained immunity model in detail. Abbreviations: Me: methylation; AC: acetylation; oxLDL: oxidized low-density lipoprotein; LPS: lipopolysaccharides; CD36: cluster of differentiation 36; TLR4: Toll-like receptor 4; NLRP3: Nod-like receptor family pyrin domain-containing 3; cROS: cytosolic reactive oxygen species; Akt: protein kinase B; mTOR: mechanistic target of rapamycin; ATP: adenosine triphosphate; GLUT1: glucose transporter 1; PTEN KO: phosphatase and tensin homology knockout; acetyl-CoA: acetyl coenzyme A; HAT: histone acetyltransferases; HMGCAR: 3-hydroxy-3-methylglutaryl CoA reductase; ACLY: ATP citrate lyase; H3K27: histone 3 lysine 27 acetylation; H314K: histone 3 lysine 14 acetylation; CBS: cystathionine-*β*-synthase; BHMT: betaine–homocysteine methyltransferase; MAT1A: methionine adenosyltransferase 1A; SAM: S-adenosylmethionine; SAH: S-adenosylhomocysteine; GNMT: glycine N-methyltransferase; PEMT: phosphatidylethanolamine N-methyltransferase; PC: phosphatidylcholine; CH_3_: methyl group.

We attempted to integrate all the findings presented here, our papers, and others' reports and proposed a novel working model of multiple-hit TI model (Figure 7) with enhanced inflammation for NASH/NAFLD development with a synergy between hyperlipidemia induced by HFD feeding and hypomethylation induced by nutrient and gene deficiencies related to methionine-homocysteine circle as we reported [84–91]. Hyperlipidemia and NAFLD are highly associated [1, 92–96]. HFCD model upregulated significantly TI enzymes and lipid peroxidation enzymes but did not significantly upregulate cytokines, chemokines, and canonical and noncanonical inflammasome regulators. Of note, most NAFLD mouse models have hyperlipidemia component (HFD) [45, 46, 97] (Figure 7(c)). Mechanistically, hyperlipidemia induced by HFD feeding acts as the first stimulation for TI [98, 99] (Figure 7(a)) via lysoPC stimulation [27], oxidized low-density lipoprotein (oxLDL) binding to CD36 [100], TLR4 [5]/TLR2 [101] and nucleotide-binding domain, leucine-rich-containing family, pyrin domain-containing 3 (NLRP3) inflammasomes [6, 26, 99, 102, 103] (Figure 7(b)).

Others also reported that HFD and HFD plus HCD feeding promote the progression of NASH/NAFLD [45, 97], which are well correlated with our new working model that hyperlipidemic stimulations are functional as the first stimulation and second stimulation in TI to enhance innate immune responses. Very interestingly, we found that methionine deficiency diet, choline deficiency diet in MCD model, and MAT1A-KO all lead to S-adenosylmethionine (SAM) decrease or deficiency [104] in methionine-homocysteine cycle [91]. Furthermore, since glycine methylation is one of the reactions that contribute most to total transmethylation flux [105], GNMT1-KO lead to decreased glycine methylation [91] and global DNA hypomethylation [106]. Since SAM is cellular methyl donor we reported [85, 87–91, 107] and reviewed [84, 108], the SAM decrease/deficiency and weakened SAM function lead to decreased ratios of SAM/S-adenosylhomocysteine (SAH), cellular hypomethylation, decreased histone methylation, and consequently increased histone acetylation, presumably including TI-related histone 3 lysine 27 acetylation (H3K27ac) and H3K14ac, as we reviewed [26, 28] and reported [27, 29, 30, 109].

Furthermore, increased histone acetylation by inhibiting histone deacetylase-2 (HDAC2) promotes macrophage infiltration and progression of NASH [110]. MAT1A and GNMT are relatively liver-specific enzymes; hypomethylation in the liver of MAT1A-KO and GNMT-KO can be striking. Hypomethylation may be an essential factor for liver injury but not essentially required for the establishment of TI since H3K4me3 also mediates TI [26] [111]. In support of our conclusion, it was reported that both hypermethylation and hypomethylation of lipid metabolism genes are found in obese patients with hypercholesterolemia [112]. Since hyperlipidemia can act alone to promote NASH/NAFLD progression and presumably the establishment of TI, both hyperlipidemia and hypomethylation act as second stimuli for TI (Figure 7(c)). As we indicated [27], the TI is a novel mechanism for qualifying any stimuli in the environments and underlying how chronic metabolic diseases [27, 28, 113] such as inflammation progression in NASH/NAFLD. Interestingly, we demonstrated that caspase-1 and caspase-11/4 not only serve as metabolic stress-derived DAMP sensors and inflammation initiators but also serve as upstream master regulators for at least partially upregulating inflammatory cytokines, chemokines, canonical and noncanonical inflammasome regulators, TI enzymes, and lipid peroxidation enzymes.

One limitation of the current study is that, due to the low-throughput nature of verification techniques in all the research laboratories, we could not verify every result we identified [61, 114]. We acknowledge that carefully designed *in vitro* and *in vivo* experimental models will be needed to verify all the findings. Nevertheless, our findings provide novel insights on the roles of proinflammatory cytokines and chemokines [58, 59] and canonical secretome [61, 115, 116], canonical and noncanonical inflammasome pathways, TI enzymes, and lipid peroxidation enzymes in promoting NASH/NAFLD progression as well as novel targets for the future therapeutic interventions for NASH/NAFLD, metabolic diseases, transplantation, and cancers.

## Figures and Tables

**(a):** 

	Human		Mouse			
GEO ID	GSE17470	GSE63067	GSE35961	GSE63027	GSE63027	GSE53381
Comparison	NASH vs. healthy	NASH vs. healthy	MCD+HFD	GNMT-KO	MAT1A-KO	HFCD
Pathways						
Serotonin degradation	↑					↑
Ethanol degradation II	↑					
Synaptic long-term depression	↑					
Noradrenaline and adrenaline degradation	↑					
Sperm motility	↑					
Phospholipases	↑					
LXR/RXR activation	↑					↑
Fatty acid *β*-oxidation I	↑			↑		↑
Nicotine degradation II	↑				↑	↑
eNOS signaling	↑					
Leukocyte extravasation signaling	↑	↑	↑			
p70S6K signaling	↑					
Retinol biosynthesis	↑					
Stearate biosynthesis I (animals)	↑			↑		
Histamine degradation	↑					
Aldosterone signaling in epithelial cells	↑					
HIPPO signaling	↑					
cAMP-mediated signaling	↑					
Synaptic long-term potentiation	↑					
Fc*γ* receptor-mediated phagocytosis in macrophages and monocytes	↑		↑			
IL-8 signaling		↑	↑			
Rac signaling			↑			
NF-*κ*B activation by viruses			↑			
Salvage pathways of pyrimidine ribonucleotides			↑			
G*α*q signaling			↑			
Pyridoxal 5′-phosphate salvage pathway			↑			
GP6 signaling pathway			↑			
Colorectal cancer metastasis signaling			↑			
ILK signaling			↑			
Integrin signaling			↑			
mTOR signaling			↑			
Phospholipase C signaling			↑			
Cardiac hypertrophy signaling (enhanced)		↑	↑			
ERK5 signaling			↑			
Sphingosine-1-phosphate signaling			↑			
Small cell lung cancer signaling			↑			
G*α*12/13 signaling			↑			
Signaling by rho family GTPases			↑			
Superpathway of cholesterol biosynthesis					↑	
PPAR signaling						
Nicotine degradation III					↑	
Melatonin degradation I						
Superpathway of melatonin degradation						
Xenobiotic metabolism CAR signaling pathway						
Xenobiotic metabolism PXR signaling pathway						
Cholesterol biosynthesis I					↑	
Cholesterol biosynthesis II (via 24,25-dihydrolanosterol)						
Cholesterol biosynthesis III (via desmosterol)					↑	

**(b):** 

	Human		Mouse			
GEO ID	GSE17470	GSE63067	GSE35961	GSE63027	GSE63027	GSE53381
Comparison	NASH vs. healthy	NASH vs. healthy	MCD+HFD vs. NCD	GNMT-KO vs. WT	MAT1A-KO vs. WT	HFCD vs. NCD
Pathways						
Superpathway of geranylgeranyl diphosphate biosynthesis I (via mevalonate)						
Estrogen biosynthesis					↑	
Acetone degradation I (to methylglyoxal)					↑	
Mevalonate pathway I					↑	
Tryptophan degradation III (eukaryotic)						
Antioxidant action of vitamin C				↑		
Oxidative phosphorylation				↑		
Glutaryl-CoA degradation				↑		
Valine degradation I				↑		
Isoleucine degradation I				↑		
RhoA signaling				↑		
Mitotic roles of polo-like kinase				↑		
Triacylglycerol biosynthesis				↑		
TCA cycle II (eukaryotic)				↑		
Role of CHK proteins in cell cycle checkpoint control				↑		
Sumoylation pathway				↑		
Apelin adipocyte signaling pathway		↑		↑		
Glutathione-mediated detoxification				↑		
Tetrahydrofolate salvage from 5,10-methenyltetrahydrofolate				↑		
CMP-N-acetylneuraminate biosynthesis I (eukaryotes)				↑		
EIF2 signaling				↑		
tRNA charging				↑		
Folate transformations I				↑		
Endocannabinoid cancer inhibition pathway					↑	
Pyrimidine ribonucleotide interconversion					↑	
Pyrimidine ribonucleotide de novo biosynthesis					↑	
NAD salvage pathway II					↑	
PPAR*α*/RXR*α* activation					↑	
NRF2-mediated oxidative stress response		↑			↑	
Glutathione redox reactions I					↑	
Pancreatic adenocarcinoma signaling					↑	
Estrogen-mediated S-phase entry					↑	
Type II diabetes mellitus signaling					↑	
PCP pathway					↑	
PDGF signaling					↑	
NF-*κ*B signaling		↑				
Neuroinflammation signaling pathway		↑				
Tec kinase signaling		↑				
B cell receptor signaling		↑				
Osteoarthritis pathway		↑				
Th17 activation pathway		↑				
Role of pattern recognition receptors in recognition of bacteria and viruses		↑				
Apelin endothelial signaling pathway		↑				
Cholecystokinin/gastrin-mediated signaling		↑				
T cell exhaustion signaling pathway		↑				
Endothelin-1 signaling		↑				
B cell activating factor signaling		↑				
RANK signaling in osteoclasts		↑				
Toll-like receptor signaling		↑				
Renin-angiotensin signaling		↑				

**(a):** 

	Patients		Mouse			
GEO ID	GSE17470	GSE63067	GSE35961	GSE63027	GSE63027	GSE53381
Comparison	NASH vs. healthy	NASH vs. healthy	MCD+HFD vs. NCD	MAT1A-KO vs. WT	GNMT-KO vs. WT	HFCD vs. NCD
Gene symbol						
ACSS1			4.47			-2.67
ACSS2			-2.55	-1.62		3.45
ADH7	2.76		1.64	1.48		1.39
AHD1A	7.97					
ADH1B	2.82					
ADH1C	4.09					
ALDH1A3			6.34			
ALDH1B1	4.38		1.62	2.24	-1.63	
ALDH2	6.33		-1.70	-1.50		1.59
ALDH3A2	2.03		2.05	-2.87	5.86	
ALDH3B1	2.49					
ALDH7A1	3.81		-4.07			1.65
ALDOA			3.69			
ALDOC	2.21		1.64	1.32		3.96
DLAT	-2.86		1.40	-2.34	1.40	1.36
ENO3	3.93	4.11				
FBP2			3.13			
HK1			2.73			
HK2					2.10	
HKDC1	-1.85		5.36	1.60		
LDHA	-1.79					
LDHB			4.99			
PCK2	7.05		4.38	-1.33		
PDHA1					1.91	
PDHB			-1.39	-1.72		
PFKFB1	3.64				1.63	1.62
PFKFB4			2.69	1.62		
PFKP			7.30			-2.27
PGAM1					1.44	
PGK1	-6.50		1.75			
PGM1		1.68	2.70	1.71		-1.64
PKM			5.27			-2.76
ADH4	41.77		-2.88	-1.51	-2.01	
ADH5	4.73		-1.33			1.45
ADH6	5.42		-4.71			
ADPGK	-2.07		-1.82			
ALDH3B2						-1.67
ALDH9A1			-1.35			1.92
ALDOB	2.05	1.50	-1.66			1.30
BPGM			-1.32	-1.37		1.32
DLD	2.58		-2.02			1.68
ENO1			-1.55		1.36	
ENO2						-2.13
FBP1	2.24		-2.23			1.49
G6PC				-5.06	-1.59	
GALM	2.16		-1.72			1.37
GAPDH			-1.61			
GCK	17.03		-33.70			
GPI	1.95					
HK3			2.71			-3.37
LDHC			-2.04			
PANK1				-1.39		1.77
PCK1			-1.88	-1.45		
PFKFB2			1.74			
PFKFB3		1.982	-1.65			-2.48
PFKL	1.738		-1.37			
PFKM			-1.98	-1.49	-1.31	
PGAM2	22.09		-2.02			
PGM2						1.52
PKLR		1.738		-1.80		
SLC2A2	9.160		-1.76			1.36
% up	25/71 (35.2%)	5/71 (7%)	20/71 (28.2%)	6/71 (8.5%)	7/71 (9.9%)	17/71 (23.9%)
% down	5/71 (7%)	0/71 (0%)	24/71 (33.8%)	13/71 (18.3%)	4/71 (5.6%)	8/71 (11.3%)

**(b):** 

	Human		Mouse			
GEO ID	GSE17470	GSE63067	GSE35961	GSE63027	GSE63027	GSE53381
Comparison	NASH vs. healthy	NASH vs. healthy	MCD+HFD vs. NCD	MAT1A-KO vs. WT	GNMT-KO vs. WT	HFCD vs. NCD
Gene symbol						
BDH1			2.33	1.89		
ACSS1			4.47			-2.67
ALDH2	6.33		-1.70	-1.50		1.59
ACSS2			-2.55	-1.62		3.45
ACAA2	2.83		-1.51		1.38	1.49
HADH		1.50			1.37	
ADH1B	2.82					
GOT1						1.79
ACO1			-1.41			1.45
ACLY			-2.40	-1.36		1.75
GLS			3.27			-2.40
IDH1	2.83		-1.40	-1.45		1.60
GLUD1			-1.55			
% up	4/24 (16.6%)	1/24 (4.16%)	3/24 (12.5%)	1/24 (4.2%)	2/24 (8.3%)	7/24 (29.2%)
% down	0/24 (0%)	0/24 (0%)	7/24 (29.2%)	4/24 (16.7%)	0/24 (0%)	2/24 (8.3%)

**(c):** 

	Human		Mouse			
GEO ID	GSE17470	GSE63067	GSE35961	GSE63027	GSE63027	GSE53381
Comparison	NASH vs. healthy	NASH vs. healthy	MCD+HFD vs. NCD	MAT1A-KO vs. WT	GNMT-KO vs. WT	HFCD vs. NCD
Gene symbol						
IDI1			2.778	1.57		10.974
IDI2	-1.655					
HMGCS1	-2.440	1.895	2.64			7.177
MVD	-1.795		4.003		1.381	5.819
MVK	4.096		5.451	1.858		3.895
HMGCR			2.091	1.764		2.663
ACAT1			2.135	1.374	1.528	
PMVK			1.45			3.366
ACAT2					1.63	
HMGCS2	7.434		-1.908	-1.738		1.681
% up	2/10 (20%)	1/10 (10%)	7/10 (70%)	4/10 (40%)	3/10 (30%)	7/10 (70%)
% down	3/10 (30%)	0/10 (10%)	1/10 (10%)	1/10 (10%)	0/10 (0%)	0/10 (0%)

**Table:** (a) COX lipid peroxidation enzymes

GEO ID	GSE35961	GSE63027	GSE63027	GSE53381
Comparison	MCD+HFD vs. NCD	MAT1A-KO vs. WT	GNMT-KO vs. WT	HFCD vs. NCD
Gene symbol				
COX10				1.391
COX11				1.388
COX14				1.303
COX15				1.372
COX17	1.302			1.364
COX18	-1.368			1.477
COX19			1.438	
COX4I1	-1.314			
COX5A	-1.584			
COX5B	-1.524			1.344
COX6A1	-1.598			1.387
COX6A2	1.378			-2.695
COX6B1	-1.437			
COX6B2		2.135	1.923	1.409
COX6C	-1.334			
COX7A1		-1.730	1.439	
COX7A2	-1.313			
COX7A2L				
COX7C		1.356		
COX8C	-1.761			
% up	2/26 (7.7%)	2/26 (7.7%)	3/26 (11.5%)	9/26 (34.6%)
% down	9/26 (34.6%)	1/26 (3.8%)	0/26 (0%)	1/26 (3.8%)

**Table:** (b) Hepatocyte glutathione peroxidase lipid antioxidant enzyme

GEO ID	GSE35961	GSE63027	GSE63027	GSE53381
Comparison	MCD+HFD vs. NCD	MAT1A-KO vs. WT	GNMT-KO vs. WT	HFCD vs. NCD
Gene symbol				
GPX3	8.857	2.387		
GPX4	1.832		1.590	
GPX7	3.763	2.196		
GPX8	2.266			-1.693
% up	4/8 (50%)	2/8 (25%)	1/8 (12.5%)	0/8 (0%)
% down	0/8 (0%)	0/8 (0%)	0/8 (0%)	1/8 (12.5%)

**Table:** (c) Cytochrome P450 CYP lipid peroxidation antioxidant (anti-inflammatory) enzymes

GEO ID	GSE35961	GSE63027	GSE63027	GSE53381
Comparison	MCD+HFD vs. NCD	MAT1A-KO vs. WT	GNMT-KO vs. WT	HFCD vs. NCD
Gene symbol				
CYP11A1	-2.023			
CYP11B2	-3.851		-1.459	
CYP17A1	-2.781		7.210	
CYP1B1	3.080			1.496
CYP20A1	2.293			
CYP21A2				
CYP2B10			-3.160	12.086
CYP2B13	9.455	3.648		7.655
CYP2B9	48.303	6.811		4.811
CYP2C29	-4.350	-2.577	-1.736	1.663
CYP2C37	-2.233	-3.070	-2.395	1.834
CYP2C38	-1.531	-2.784	2.694	2.499
CYP2C39	2.771			1.751
CYP2C40				1.830
CYP2C54	-113.732	-1.679	-3.681	1.907
CYP2C55		1.681	-2.085	4.526
CYP2C65				1.413
CYP2F1				
CYP2J11			1.347	1.621
CYP2J2				
CYP2J5	-4.063		-1.569	1.442
CYP2R1	-2.226	-2.032		1.441
CYP2S1				-1.756
CYP2U1	-6.007		-1.990	1.473
CYP2W1				
CYP3A7				
CYP4A10				2.235
CYP4A11				
CYP4A12A	-7.436	3.664	1.670	2.931
CYP4A12B				2.789
CYP4A14	3.575	-13.392	16.111	14.158
CYP4A31		-2.773		13.473
CYP4B1	-1.598	-1.619		
CYP4F13	-2.811			1.525
CYP4F18				
% up	6/44 (13.6%)	4/44 (9.1%)	5/44 (11.4%)	22/44 (50%)
% down	13/44 (29.5%)	8/44 (18.2%)	8/44 (18.2%)	1/44 (2.3%)

**Table:** (d) Arachidonic acid metabolism enzymes

GEO ID	GSE35961	GSE63027	GSE63027	GSE53381
	MCD+HFD vs. NCD	MAT1A-KO vs. WT	GNMT-KO vs. WT	HFCD vs. NCD
Gene symbol				
CYP2C37	-2.233	-3.070	-2.395	1.834
CYP2C29	-4.350	-2.577	-1.736	1.663
CYP2C54	-113.732	-1.679	-3.681	1.907
CYP2J5	-4.063		-1.569	1.442
CYP4F13	-2.811			1.525
CYP2U1	-6.007		-1.990	1.473
PLA2G4C	-3.499			
LTA4H	-2.953			
CYP 20	-1.818			1.432
CYP4A12A	-7.436	3.664	1.670	2.931
CYP2C38	-1.531	-2.784	2.694	2.499
CYP2C65				1.413
PLA2G6				1.429
ALOX12B				1.439
PTGES2				1.508
CYP2J11			1.347	1.621
CYP2C40				1.830
CYP4A10				2.235
CYP4A12B				2.789
CYP2C55		1.681	-2.085	4.526
CYP2B10			-3.160	12.086
CYP4A31		-2.773		13.473
CYP4F18				
CYP2C39	2.771			1.751
CYP4A14	3.575	-13.392	16.111	14.158
CYP2B13	9.455	3.648		7.655
CYP2B9	48.303	6.811		4.811
% up	4/26 (15.4%)	4/26 (15.4%)	4/26 (15.4%)	24/26 (92.3%)
% down	11/26 (42.3%)	6/26 (23.1%)	7/26 (26.9%)	0/26 (0%)

**(a):** 

GEO ID	GSE115094	GSE32515
Comparison	Caspase-11-KO vs. WT	Caspase-1-KO vs. WT
Gene symbol		
CXCL10	1.98	
CCL20	5.07	
EDN1	3.45	
CXCL16	2.50	
CKLF	2.83	
CCL5	4.81	
TIMP1	10.52	
CCL2	4.75	
Ccl7	5.99	
TNF	2.72	
TSLP	7.42	
CCL8	2.70	
IL27	4.15	
PF4	4.82	
CCL17	13.15	
CCL3	3.58	
CCL4	7.09	
CXCL5	4.50	
CXCL14		3.219
DKK 3	-5.54	
CX3CL1	-4.49	
CXCL12	-3.50	
CSF1	-3.04	
TNFSF10	-2.23	
NAMPT	-2.08	
WNT5A	-1.79	
SPP1	-5.87	
LTB		-1.551
% up	18/39 (46.2%)	1/39 (2.6%)
% down	8/39 (20.5)	1/39 (2.6%)

**(b):** 

Canonical inflammasome pathway regulators upregulated in NASH		
GEO ID	GSE115094	GSE32515
Comparison	Caspase-11-KO vs. WT	Caspase-1-KO vs. WT
Gene symbol		
TXN2	1.749	
PYCARD	2.469	
Naip5	1.602	
Naip2	1.895	
CYBA	3.599	
PRKCD	-1.643	
ANTXR2	-3.523	
CTSB	-2.328	
NAMPT	-2.083	
RIPK1	-1.754	
% up	5/28 (17.9%)	0/28 (0%)
% down	5/28 (17.9%)	0/28 (0%)

**(c):** 

Trained immunity	GEO ID	GSE115094	GSE32515
Pathways	Comparison	Caspase-11-KO vs. WT	Caspase-1-KO vs. WT
Glycolysis	LDHB	2.520	
	PFKFB3	1.844	
	ALDOA	1.883	
	FBP1	4.254	
	ALDOA	1.883	
	ADH7		1.624
	PFKFB1		1.511
	PFKFB2		1.606
	HK1	-2.535	
	HK2	-4.554	
	HKDC1	-1.550	
	PDHA1	-3.277	
	PFKFB4	-1.570	
	PFKP	-1.544	
	PGAM1	-2.484	
	ENO1	-1.689	
	DLAT	-1.770	
	GALM	-1.700	
	PANK1	-1.892	
	PFKL	-4.133	
	PGM2	-1.663	
	PKLR	-3.863	-2.354
	ALDH1B1	-2.881	
	ALDH2	-2.171	
	ALDH3A2	-1.840	
	DLAT	-1.770	
	ACSS2		-1.836
	ADH4		-2.019
	GCK		-3.334
	SLC2A2		-1.789
	ACSS1	-3.206	
	ALDH1B1	-2.881	
	ALDH2	-2.171	
	ALDH3A2	-1.840	
Mevalonate	PMVK	1.699	
	IDI1	-3.248	
	HMGCS1	-1.818	
	HMGCR	-1.894	
	ACAT1	-1.917	
	IDI1	-3.248	
Acetyl-CoA generation	GOT1	-1.684	1.701
	BDH1	-1.964	
	ACO1	-1.601	
	ACLY	-2.203	-2.33
	% up	6/68 (8.8%)	4/68 (5.9%)
	% down	31/68 (45.6%)	6/68 (8.8%)

**(d):** 

Lipid peroxidation	GEO ID	GSE115094	GSE32515
Enzymes	Comparison	Caspase-11-KO vs. WT	Caspase-1-KO vs. WT
COXs	COX14	5.806	
	COX18	1.862	
	COX5B	6.309	
	COX6A1	5.582	
	COX6B2	12.677	
	COX7A1	11.346	
	COX7C	11.363	
	COX19	4.005	
	COX15	-3.650	
CYPs	CYP2R1	4.459	
	CYP2B10	7.468	4.070
	CYP2C39		15.441
	CYP4A31		1.831
	CYP2U1	-2.833	
	CYP1B1	-2.058	
	CYP20A1	-2.466	
	CYP2B9		-3.075
	CYP2C37		-6.050
	CYP2C38		-3.200
	CYP2C54		-2.609
	CYP2C55		-6.187
	CYP4F18		-1.575
GPX	GPX4	6.696	
	GPX7	3.769	
	GPX8	3.769	
	GPX3	-1.549	
AA metabolism	PLA2G6	-2.293	
	% up	13/46 (28.3%)	3/46 (6.5%)
	% down	6/46 (13%)	6/46 (13%)

## Data Availability

The 10 microarray and/or RNA-seq expression data were collected from the National Institutes of Health- (NIH-) National Center for Biotechnology Information- (NCBI-) Gene Expression Omnibus (GEO) databases (GSE63067, GSE17470, GSE35961, GSE63027, GSE53381, GSE115094, GSE32515, and GSE24187).

## Supplementary Materials

Supplementary Materials Supplementary figures and tables provide the following: (1) housekeeping gene expression data used for quality control, (2) description, GEO ID, and PMID for microarray and RNA-seq datasets, and (3) Ingenuity Pathway Analysis (IPA) for all 6 NASH datasets and 7 trained immunity gene list.
