# Supplementary material for: Hyperlipidemia May Synergize with Hypomethylation in Establishing Trained Immunity and Promoting Inflammation in NASH and NAFLD
Source: J Immunol Res. 2021 Nov 23;2021:3928323. doi: 10.1155/2021/3928323 (PMC8632388; doi:10.1155/2021/3928323)

Supplemental Figure 1. The top 20 pathways of IPA analysis in **(A)** two human NASH and **(B)** four mouse NASH. The detailed information are showed in Table 1.

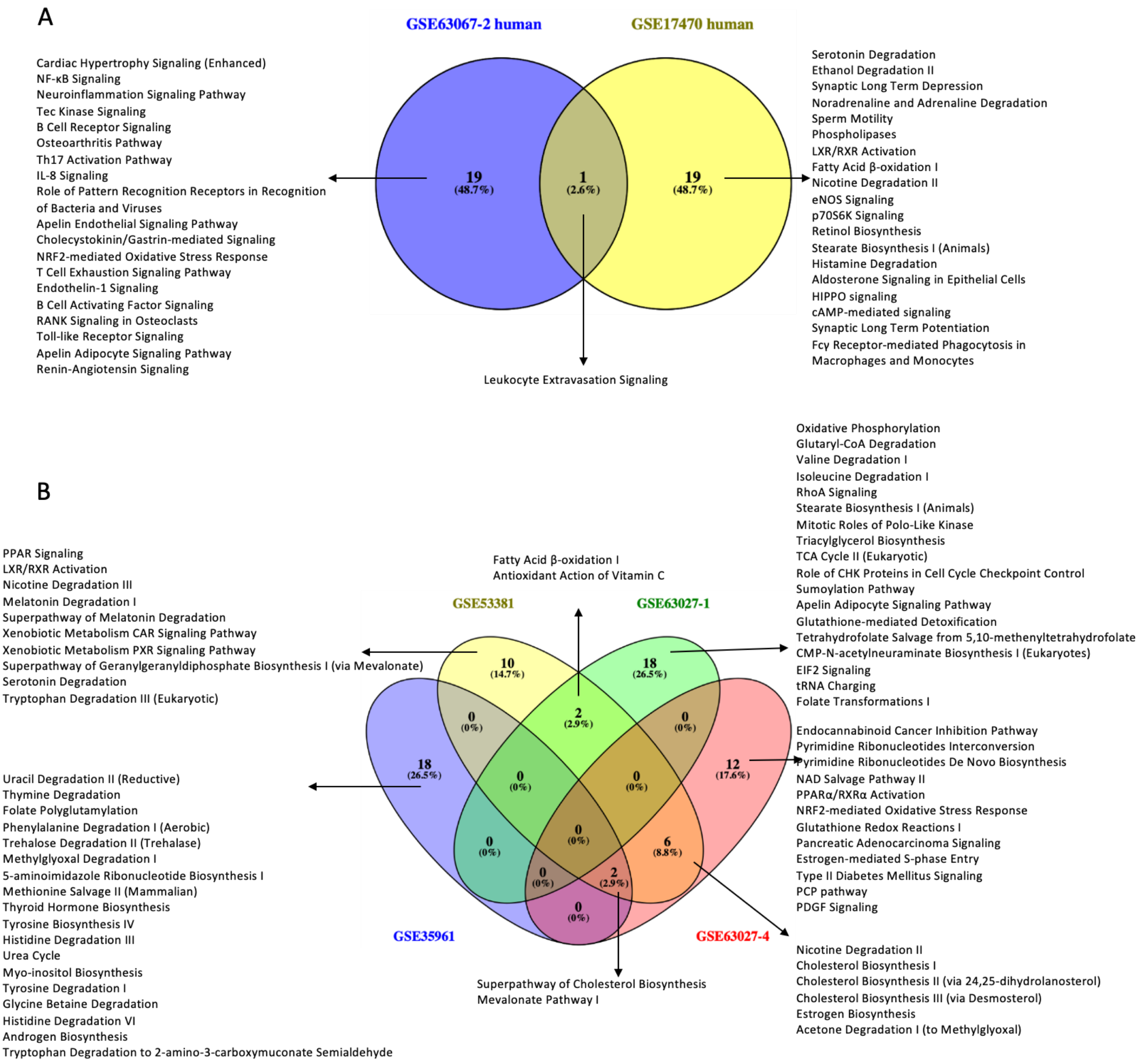

Supplement: Supplementary Materials — Supplementary figures and tables provide the following: (1) housekeeping gene expression data used for quality control, (2) description, GEO ID, and PMID for microarray and RNA-seq datasets, and (3) Ingenuity Pathway Analysis (IPA) for all 6 NASH datasets and 7 trained immunity gene list. [file 3928323.f1.zip › Supplementary Figure 1 (1).pdf]
