# Supplementary material for: Hyperlipidemia May Synergize with Hypomethylation in Establishing Trained Immunity and Promoting Inflammation in NASH and NAFLD
Source: J Immunol Res. 2021 Nov 23;2021:3928323. doi: 10.1155/2021/3928323 (PMC8632388; doi:10.1155/2021/3928323)
Supplement: Supplementary Materials — Supplementary figures and tables provide the following: (1) housekeeping gene expression data used for quality control, (2) description, GEO ID, and PMID for microarray and RNA-seq datasets, and (3) Ingenuity Pathway Analysis (IPA) for all 6 NASH datasets and 7 trained immunity gene list. [file 3928323.f1.zip › Supplementary Table 1 (1).pdf]

Supplemental Table 1. House keeping genes (HKG) for datasets in this study. **(A)** Human NASH, **(B)** Mouse NASH/NAFLD models, C) NASH/NAFLD Datasets.

A

	GSE63067		GSE17470	
	NASH vs Healthy		NASH vs Healthy	
HKG Symbol	*P* value	FC	*P* value	FC
RRN18S				
ACTB	0.60	-1.06	0.57	1.20
GAPDH	0.37	1.07	0.86	-1.06
PGK 1				
PPIA	0.17	-1.10		
RPL13A			0.29	-1.57
RPLP0	0.14	-1.10		
ARBP				
B2M	0.44	1.04	0.37	-1.28
YWHAZ	0.20	1.11	0.12	-1.54
SDHA	0.07	1.14	0.73	1.09
TFRC	0.46	-1.22	0.21	-3.60
GUSB	0.75	1.03	≤0.001	3.59
HMBS	0.02	-1.23	0.82	-1.08
HPRT1	0.25	-1.21	0.56	-1.34
TBP	0.30	1.06	0.46	-1.16

B

	GSE35961		GSE63027		GSE63027	
	MCD+HFD vs NCD		GNMT-KO vs WT		MATA1-KO vs WT	
HKG Symbol	*P* Value	FC	*P* Value	FC	*P* Value	FC
RRN18S						
ACTB	≤0.001	-1.85	0.83	-1.01	0.76	-1.07
GAPDH	≤0.001	-1.77	0.04	1.14	0.07	-1.13
PGK 1.00						
PPIA						
RPL13A	0.25	-1.11	0.04	-1.24	≤0.001	-1.71
RPLP0	0.43	-1.07	0.37	1.05	0.54	-1.04
ARBP						
B2M	≤0.001	-1.91	0.68	-1.02	0.14	1.08
YWHAZ	0.65	-1.05	0.09	-1.18	0.72	-1.02
SDHA	0.36	-2.26	0.33	-1.10	0.01	-1.38
TFRC	0.19	1.55	0.78	-1.04	0.11	1.11
GUSB	0.10	1.27	0.75	1.04	0.45	-1.09
HMBS	0.87	1.02	≤0.001	1.36	0.58	1.04
HPRT1						
TBP	0.34	-2.07	0.51	1.06	0.10	-1.09
