# Supplementary material for: Hyperlipidemia May Synergize with Hypomethylation in Establishing Trained Immunity and Promoting Inflammation in NASH and NAFLD
Source: J Immunol Res. 2021 Nov 23;2021:3928323. doi: 10.1155/2021/3928323 (PMC8632388; doi:10.1155/2021/3928323)
Supplement: Supplementary Materials — Supplementary figures and tables provide the following: (1) housekeeping gene expression data used for quality control, (2) description, GEO ID, and PMID for microarray and RNA-seq datasets, and (3) Ingenuity Pathway Analysis (IPA) for all 6 NASH datasets and 7 trained immunity gene list. [file 3928323.f1.zip › Supplementary Table 2 (1).pdf]

**Supplementary Table 2A. NASH/NAFLD Datasets.**

| | GEO ID | NAFLD Status | COMPARISON | Tissue | Reference (PMID) |
|---|---|---|---|---|---|
| Mouse | GSE35961 | NASH | Methionine/choline deficient (MCD) + HFD vs NCD | Liver | 23028442 |
| | GSE63027 | | glycine N-methyltransferase knockout (GNMT-KO) vs WT | Liver | 25993042 |
| | GSE63027 | | methionine adenosyl transferase knockout (MAT1A-KO) vs WT | Liver | 25993042 |
| | GSE53381 | | High Fat with Cholesterol | Liver | 25310404 |
| Human | GSE63067 | NASH | NASH vs Healthy | Liver | 25993042 |
| | GSE17470 | | NASH vs Healthy | Liver | 20221393 |

**Supplementary Table 2B. Mouse KO Mechanism Datasets.**

| | GEO ID | NAFLD Status | COMPARISON | Tissue | Reference (PMID) |
|---|---|---|---|---|---|
| Mouse | GSE115094 | Caspase11-KO vs WT | Caspase11-KO vs WT | Illeum | 30021146 |
| | GSE32515 | Caspase 1-KO vs WT | Caspase 1-KO vs WT | Liver | 23160218 |
| Human | GSE24187 | Mevalonate Trained Immunity | Rosuvastatin vs Untreated (Human Primary Hepatocytes ) | Liver | 21869732 |
| | GSE24187 | | Atorvastatin vs Untreated (Human Primary Hepatocytes ) | Liver | 21869732 |
