# Supplementary material for: Hyperlipidemia May Synergize with Hypomethylation in Establishing Trained Immunity and Promoting Inflammation in NASH and NAFLD
Source: J Immunol Res. 2021 Nov 23;2021:3928323. doi: 10.1155/2021/3928323 (PMC8632388; doi:10.1155/2021/3928323)
Supplement: Supplementary Materials — Supplementary figures and tables provide the following: (1) housekeeping gene expression data used for quality control, (2) description, GEO ID, and PMID for microarray and RNA-seq datasets, and (3) Ingenuity Pathway Analysis (IPA) for all 6 NASH datasets and 7 trained immunity gene list. [file 3928323.f1.zip › Supplementary Table 3 (1).pdf]

Supplemental Table 3a. The 71 glycolytic enzymes are shown in the list.

Gene symbol	Gene name
ACSS1	acyl-CoA synthetase short chain family member 1
ACSS2	acyl-CoA synthetase short chain family member 2
ADH1A	alcohol dehydrogenase 1A (class I), alpha polypeptide
ADH1B	alcohol dehydrogenase 1B (class I), beta polypeptide
ADH1C	alcohol dehydrogenase 1C (class I), gamma polypeptide
ADH4	alcohol dehydrogenase 4 (class II), pi polypeptide
ADH5	alcohol dehydrogenase 5 (class III), chi polypeptide
ADH6	alcohol dehydrogenase 6 (class V)
ADH7	alcohol dehydrogenase 7 (class IV), mu or sigma polypeptide
ADPGK	ADP dependent glucokinase
AKR1A1	aldo-keto reductase family 1 member A1
ALDH1A3	aldehyde dehydrogenase 1 family member A3
ALDH1B1	aldehyde dehydrogenase 1 family member B1
ALDH2	aldehyde dehydrogenase 2 family member
ALDH3A1	aldehyde dehydrogenase 3 family member A1
ALDH3A2	aldehyde dehydrogenase 3 family member A2
ALDH3B1	aldehyde dehydrogenase 3 family member B1
ALDH3B2	aldehyde dehydrogenase 3 family member B2
ALDH7A1	aldehyde dehydrogenase 7 family member A1
ALDH9A1	aldehyde dehydrogenase 9 family member A1
ALDOA	aldolase, fructose-bisphosphate A
ALDOB	aldolase, fructose-bisphosphate B
ALDOC	aldolase, fructose-bisphosphate C
BPGM	bisphosphoglycerate mutase
DLAT	dihydrolipoamide S-acetyltransferase
DLD	dihydrolipoamide dehydrogenase
ENO1	enolase 1
ENO2	enolase 2
ENO3	enolase 3
FBP1	fructose-bisphosphatase 1
FBP2	fructose-bisphosphatase 2
G6PC	glucose-6-phosphatase catalytic subunit
G6PC2	glucose-6-phosphatase catalytic subunit 2
GALM	galactose mutarotase
GAPDH	glyceraldehyde-3-phosphate dehydrogenase
GAPDHS	glyceraldehyde-3-phosphate dehydrogenase, spermatogenic
GCK	glucokinase
GPI	glucose-6-phosphate isomerase
HK1	hexokinase 1
HK2	hexokinase 2
HK3	hexokinase 3
HKDC1	hexokinase domain containing 1
LDHA	lactate dehydrogenase A
LDHAL6A	lactate dehydrogenase A like 6A
LDHAL6B	lactate dehydrogenase A like 6B
LDHB	lactate dehydrogenase B
LDHC	lactate dehydrogenase C
PANK1	pantothenate kinase 1
PCK1	phosphoenolpyruvate carboxykinase 1
PCK2	phosphoenolpyruvate carboxykinase 2, mitochondrial
PDHA1	pyruvate dehydrogenase E1 alpha 1 subunit
PDHA2	pyruvate dehydrogenase E1 alpha 2 subunit
PDHB	pyruvate dehydrogenase E1 beta subunit
PFKFB1	6-phosphofructo-2-kinase/fructose-2,6-biphosphatase 1
PFKFB2	6-phosphofructo-2-kinase/fructose-2,6-biphosphatase 2
PFKFB3	6-phosphofructo-2-kinase/fructose-2,6-biphosphatase 3
PFKFB4	6-phosphofructo-2-kinase/fructose-2,6-biphosphatase 4
PFKL	phosphofructokinase, liver type
PFKM	phosphofructokinase, muscle
PFKP	phosphofructokinase, platelet
PGAM1	phosphoglycerate mutase 1
PGAM2	phosphoglycerate mutase 2
PGAM4	phosphoglycerate mutase family member 4
PGK1	phosphoglycerate kinase 1
PGK2	phosphoglycerate kinase 2
PGM1	phosphoglucomutase 1
PGM2	phosphoglucomutase 2
PKLR	pyruvate kinase L/R
PKM	pyruvate kinase M1/2
SLC2A2	solute carrier family 2 member 2
TPI1	triosephosphate isomerase 1

Supplemental Table 3b. The 24 acetyl-CoA generating enzymes are shown in the list.

Gene symbol	Gene name
BDH1	3-hydroxybutyrate dehydrogenase type 1
ACSS1	acyl-CoA synthetase short-chain family member 1
ALDH2	aldehyde dehydrogenase 2 family
ACSS2	acyl- CoA synthetase short-chain family member 2
ACAA2	acetyl-CoA acyltransferase 2
HADH	hydroxyacyl-CoA dehydrogenase
ADH1B	alcohol dehydrogenase IB (class I) beta polypeptide)
ACO1	aconitase 1
ACLY	ATP citrate lyase
GLS	Glutaminase
GOT1	Glutamate Oxaloacetate Transaminase 1
IDH1	isocitrate dehydrogenase 1
GLUD1	glutamate dehydrogenase 1
OXCT	3-oxoacid CoA transferase
ECH	enoyl-CoA hydratase
BCKD	branched-chain α-ketoacid dehydrogenase
BCAT2	branched chain amino-acid transaminase 2
BCAT1	branched chain amino-acid transaminase 1
ALDH1A1	aldehyde dehydrogenase 1 family member A1
ACAT2	acetyl-CoA acetyltransferase 2
ACAT1	acetyl-CoA carboxylase 1
ACAD	acyl-CoA dehydrogenase
ACAC	acetyl-CoA carboxylase
PDC	pyruvate dehydrogenase complex

Supplemental Table 3c. The 10 mevalonate enzymes are shown in the list.

Gene symbol	Gene name
ACAT1	acetyl-CoA acetyltransferase 1
ACAT2	acetyl-CoA acetyltransferase 2
MVK	mevalonate kinase
MVD	mevalonate diphosphate decarboxylase
IDI1	isopentenyl-diphosphate delta isomerase 1
IDI2	isopentenyl-diphosphate delta isomerase 2
HMGCS1	3-hydroxy-3-methylglutaryl-CoA synthase 1
HMGCS2	3-hydroxy-3-methylglutaryl-CoA synthase 2
HMGCR	3-hydroxy-3-methylglutaryl-CoA reductase
PMVK	phosphomevalonate kinase
